# Optimizing concurrent training programs: A review on factors that enhance muscle strength

**DOI:** 10.1097/MD.0000000000041055

**Published:** 2024-12-27

**Authors:** Tao Wang, Shumin Bo

**Affiliations:** a Capital University of Physical Education and Sports, Beijing, China; b School of Physical Education, Liaocheng University, Liaocheng, China.

**Keywords:** adaptation factors, concurrent training, interference effect, muscle fitness, training optimization

## Abstract

The purpose of this study is to explore the factors that should be considered when designing concurrent training programs to minimize the “interference effect.” This study provides a comprehensive summary of various perspectives from existing studies on factors such as the ability level of the target group, the frequency and intensity of concurrent training, the order and interval time of resistance and endurance training, nutritional arrangements during training, and gender differences in concurrent training. The results of this study suggest that the emergence of the “interference effect” in concurrent training is influenced by several factors, with a particular emphasis on the adaptation status of muscle fitness when compared to resistance training alone. However, it is important to note that the current understanding of this theory remains somewhat ambiguous. The ability level of participants plays a crucial role in training adaptation and the specificity of post-training skeletal muscle molecular responses. Therefore, the participant’s capacity level is likely a key determinant of the extent of the interference effect in concurrent training. In addition to training-related factors such as frequency, duration, intensity, volume, training sequence, recovery time, and nutritional intake, non-training-related variables, including the methodology used to assess various metrics, also significantly impact the design of concurrent training programs. These factors collectively influence the overall outcomes and provide insight into the extent of the “interference effect” in concurrent training. Understanding these aspects is essential for comprehending the phenomenon of the “interference effect” in concurrent training.

## 1. Introduction

Endurance and strength are crucial aspects of physical fitness and are essential for achieving male athletic excellence. The World Health Organization strongly recommends that individuals from different populations engage in appropriate endurance and muscular strength training to improve physical function, overall health, and prevent chronic diseases throughout their lives.^[1]^ However, it is important to note that aerobic and strength training elicit different biological adaptations, which may be affected when performed together.^[2]^ Therefore, when designing concurrent endurance and resistance training programs for diverse populations, it is important to consider the divergent outcomes of these biological adaptations.

Concurrent training has been widely used in high-level sports training and fitness regimens.^[3]^ However, it took time to fully understand the adaptive mechanisms involved in concurrent training. In 1980, American physiologist Hickson conducted pioneering experiments that revealed an observable impact on muscle strength gains when endurance and resistance training were performed simultaneously.^[4]^ This phenomenon, initially referred to as the “interference effect,” was attributed to the interference of endurance training with muscle strength development. As a result, this “interference effect” sparked significant research interest within the academic community. However, recent studies have provided a more nuanced perspective, suggesting that the extent of neural and muscular adaptations may depend on a complex interplay of training program variables and individual biological predispositions.^[5]^ Contrary to Hickson initial findings, contemporary research has indicated that concurrent training may not necessarily hinder neuromuscular adaptations.^[6–8]^

The exploration of training modalities to enhance endurance and muscular fitness in diverse populations, as well as their long-term adaptations, is a significant focus of academic research. Different training protocols can have different effects on training outcomes and benefits. Individuals in various populations typically aim to optimize concurrent training methods to efficiently improve aerobic and muscular fitness in a shorter period of time. When selecting concurrent training modalities, it is important to consider the optimal sequencing of endurance and resistance training, whether these activities should be performed on the same day or separately, determining appropriate intensity levels for both endurance and resistance training, establishing training frequency, investigating potential gender differences in concurrent training effects, and addressing nutritional supplementation requirements during concurrent training.

**Figure 1.**
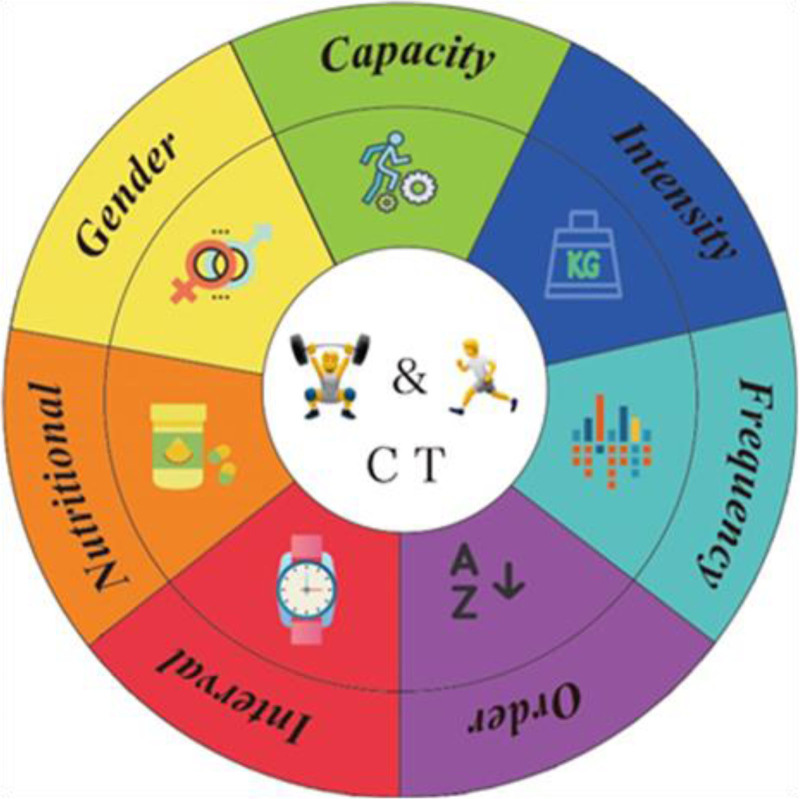
Factors to be considered in the design of concurrent training programs.

This study aims to address the above important questions (as shown in Fig. 1) and provide recommendations for the effective design of concurrent training programs in the future.

## 2. Capacity of participants

Concurrent training encompasses a diverse range of participant groups, including adolescents, older individuals, and various subgroups such as young adults, middle-aged individuals, and the elderly. Each stage exhibits distinct physiological adaptations to training. Additionally, disparities in physiological responses can be observed among healthy and unhealthy populations, males and females, different racial groups, and participants with varying levels of athletic ability.^[5]^ It is important to acknowledge that the same training program may result in varying levels of physiological adaptation among individuals and groups. Therefore, research efforts should include a wide array of participant groups to effectively generalize the results to the target population.

In the context of concurrent training, individuals who infrequently engage in exercise or training tend to exhibit similar physiological adaptive responses whether they undergo single-mode or concurrent training. The interference effect is less pronounced in this group.^[9]^ However, as the duration of the exercise stimulus increases to enhance adaptive capacity, the interference effect becomes more apparent during concurrent training. On the other hand, individuals who engage in regular training show a more significant reduction in muscle fitness when endurance and resistance training are performed concurrently compared to when they are performed alone.^[10,11]^ This distinction suggests that regular trainers have a more limited potential for adaptive mechanisms during similar workouts, requiring a more specific training regimen to achieve further performance improvements.

The cyclic training approach may be more advantageous in enhancing strength qualities in athletes compared to concurrent training.^[12]^ Although the precise mechanism is still unclear, studies suggest that regularly trained muscles show reduced expression of genes and proteins involved in anabolic processes after acute resistance training.^[13]^ On the other hand, infrequent or intermediate participants show enhanced molecular responses and muscle hypertrophy after short-term resistance training.^[14,15]^ Therefore, concurrent training programs may be more effective for populations that do not regularly engage in training or exercise. However, individuals who regularly participate in training may demonstrate more selective adaptations or even diminished adaptive responses in certain physiological aspects. Therefore, the athleticism level of the population is an important consideration when implementing concurrent training programs.

Furthermore, professional athletes typically have several years of training experience, which can potentially affect the expected physiological adaptations during concurrent training interventions. Additionally, the quality and skill requirements for athletes vary across different sports, and they often need specific training cycles to develop particular physical qualities while maintaining the relative stability of other attributes. Therefore, when implementing concurrent training programs for professional athletes in different sports, the program design should align with the specific needs of different physical qualities to achieve the desired training effects.

## 3. Training frequency

The frequency and duration of endurance training have a significant impact on the outcomes of concurrent training. Increasing the frequency and duration of endurance training in concurrent programs has been shown to result in greater improvements in body fat percentage, maximal oxygen uptake (VO_2max_), and running economy.^[16]^ Interestingly, the frequency of resistance training does not seem to have a significant effect on these metrics.^[17]^ However, it is important to note that the frequency of endurance training can have a more pronounced effect on muscle adaptation during concurrent resistance training. Research suggests that endurance training 3 times per week may hinder improvements in muscle fitness during concurrent training,^[18–20]^ while training 2 times per week seems to have a lesser impact on muscle fitness improvements in a concurrent training regimen.^[6,21]^

Previous research by Wilson and colleagues indicated a negative correlation between the development of maximal strength and the duration and frequency of endurance training.^[17]^ Specifically, longer durations of endurance training (ranging from 20–30 minutes per day to 50–60 minutes per day) and higher frequencies of endurance training (from 1–5 sessions per week) were associated with reduced improvements in maximal strength during concurrent training.^[17]^ Similarly, Jones and colleagues found that endurance training 3 times per week had a more detrimental effect on the development of strength qualities compared to endurance training once per week.^[22]^

However, it is important to note that recent studies have shown significant variations in muscular fitness improvements among individuals with different exercise habits.^[23]^ In some cases, moderate and regular exercisers have demonstrated similar improvements in muscular fitness, despite having similar endurance training durations and frequencies.^[24]^ Therefore, the relationship between endurance training frequency and strength development during concurrent training may not be universally applicable and could depend on individual factors.

Traditional methods of testing limb dimensions have led some researchers to argue that muscle hypertrophy occurs relatively slowly compared to the increase in muscle strength.^[25]^ However, modern technologies such as magnetic resonance imaging and ultrasound scanning have shown that significant muscle hypertrophy can occur after just 3 weeks of resistance training.^[26]^ Furthermore, muscle size stabilizes after approximately 3 months of consistent resistance training in young individuals with no prior training experience.^[27]^

In terms of VO_2max_, even as little as 1 week of aerobic training can lead to significant improvements. If no load adjustments are made, oxygen uptake levels tend to stabilize after 3 weeks. In untrained obese male participants, extended endurance training resulted in a 10% increase in VO_2max_ at 4 months, followed by a 7% increase from 5 to 9 months, with VO_2max_ stabilizing at 10 to 16 months without further increases; a similar trend was observed in female participants, with an 11% increase in VO_2max_ at fourth months, followed by a 10% increase from 5 to 9 months, and subsequent stabilization.^[28]^

It is clear that the frequency and duration of training in concurrent programs can result in different physiological adaptations. These differences may also be affected by individual variations. Therefore, it is important to customize the frequency and duration of endurance and resistance training in concurrent programs when scheduling them in order to achieve specific training effects.

## 4. Training intensity

The intensity of concurrent training can be determined by considering the intensity levels of both endurance training and resistance training. Endurance training intensity can be classified into low-intensity continuous training, moderate-intensity continuous training, and high-intensity interval training (HIIT), while resistance training can be designed with varying levels of intensity, including high and low intensity. Different combinations of these intensity levels for endurance and resistance training can result in various forms of concurrent training intensity. However, it is important to note that most researchers have primarily focused on the intensity of endurance training when studying the interference effect phenomenon, and there has been limited investigation into the design of resistance training programs in terms of intensity variation.

Souza et al compared the acute effects of different exercise intensities on maximal strength and strength endurance.^[29]^ Their findings indicate that higher-intensity acute aerobic training can impair muscular strength and endurance development, suggesting that the interference phenomenon may be more pronounced with chronic interventions at higher aerobic intensities. On the other hand, higher endurance intensity can provide robust cardiovascular stimulation, benefiting cardiorespiratory fitness and cardiovascular health.^[30,31]^ As a result, HIIT offers a notable advantage in enhancing cardiorespiratory fitness and metabolism compared to low-intensity continuous training and moderate-intensity continuous training.^[32,33]^ High-intensity endurance training is also significant in the training regimens of high-level athletes for improving cardiorespiratory fitness.^[34]^ However, it is important to consider that high-intensity endurance training may induce greater levels of fatigue compared to low and moderate-intensity continuous training. The accumulation of skeletal muscle fatigue, especially when concurrent with resistance training, may lead to a stronger interference effect, particularly when the timing of resistance and endurance training sessions is closely spaced.^[29,35,36]^

HIIT is commonly used by individuals who regularly engage in training or exercise. During HIIT, a greater number of motor units are activated and recruited, leading to physiological adaptations in a wider range of skeletal muscles. This aligns with the activation of fast-twitch muscle fibers during resistance training. Therefore, concurrent training with power cycling, for example, may offer advantages over concurrent training with running.^[37–39]^ Some studies have shown that concurrent training with low-intensity continuous endurance training and a combination of high-intensity endurance training can interfere with gains in muscular fitness.^[40,41]^ Silva et al conducted a study with young women, dividing them into groups undergoing moderate-intensity continuous concurrent training (running), high-intensity interval concurrent training (running), moderate-intensity continuous concurrent training (power cycling), and resistance training alone, twice a week for 11 weeks.^[42]^ The results indicated that, regardless of exercise form and intensity, all groups showed no significant differences in strength-related outcomes. This suggests that twice-weekly concurrent training over an 11-week period did not result in an interference effect among young females, thus positively impacting their health and fitness.

Another study by Fyfe et al revealed that there was no significant difference in strength quality improvement between high-intensity interval-type concurrent training and moderate-intensity continuous training-type concurrent training. However, both protocols had some impact on the development of strength quality.^[43]^

According to a review by Sabag et al, it has been emphasized that concurrent training with HIIT as an endurance training modality does not have a negative impact on muscular fitness.^[44]^ Any potential interference between resistance and endurance training can be minimized by allowing sufficient intervals between the 2 types of training sessions. Additionally, HIIT has been found to be more effective in improving cardiorespiratory fitness levels compared to moderate-intensity continuous training.^[45–47]^

Considering the various intensity levels for endurance training and their different physiological effects, especially when intensities exceed VO_2max_ levels, selecting appropriate endurance training intensities becomes a crucial factor in designing concurrent training programs.

## 5. Order of resistance and endurance training

Concurrent training, which combines both resistance and endurance training, offers a time-efficient approach to simultaneously improve cardiorespiratory fitness and muscular fitness.^[48]^ However, the acute interference hypothesis suggests that smaller concurrent training loads may not lead to interference effects. Nonetheless, residual fatigue from the first training component in a more demanding concurrent training session can negatively impact the quality and performance of the second training component. This compromised ability of the muscles to generate sufficient stimulation for the second training session creates an unfavorable molecular environment for neuromuscular adaptations, thereby diminishing the expected physiological adaptive potential.^[49]^ Consequently, the sequencing of the 2 training sessions becomes crucial in maximizing adaptations to concurrent training.

One approach is to schedule resistance training before endurance training. This sequence has been suggested to be more beneficial for lower extremity neuromuscular adaptations and muscle strength improvements.^[50,51]^ Conversely, performing endurance training before resistance training may enhance cardiorespiratory fitness and endurance exercise performance.^[52]^ The order of training may affect the mechanism of adaptation, however, some studies have found no significant sequential effects on aerobic capacity, static strength, or body fat percentage.^[29]^ Improvements in strength qualities appear to be more susceptible to interference from endurance training, while improvements in cardiorespiratory fitness and cardiovascular adaptations do not seem to be significantly affected by training sequence.^[15]^

Maximum strength plays a crucial role in athletic performance and overall daily functioning. It is particularly important to optimize the development of maximum strength for both athletes and healthy individuals. Muscle strength is closely linked to health and has a significant impact on controlling health risks and mortality.^[53,54]^ A review conducted by Murlasits et al demonstrated that a concurrent training model, where resistance training is performed before aerobic training, is more effective in developing maximal strength compared to the model where aerobic training is performed before resistance training.^[36]^ In a recent study that examined the effects of different exercise sequences on health-related fitness indices in moderately active, healthy young males, researchers found that both sequencing orders resulted in similar improvements in maximal strength, lean body mass, and VO_2max_ after a 9-week intervention. However, both sequencing orders showed less growth in the explosive strength index than the strength-only group. The participants’ subjective perceptions indicated a preference for starting the training session with resistance training.^[55]^

Many studies examining the effects of concurrent training exercise sequences on physiological adaptations and athletic performance did not include combinations of resistance or endurance training performed in isolation.^[29,56–65]^ Therefore, the lack of control in experimental studies does not directly indicate whether adaptations to endurance or resistance training are affected differently by the addition of another training modality. Additionally, the effectiveness of concurrent training may vary depending on whether the 2 training units are performed consecutively or separated by a recovery period of a few hours or even a day. In situations where recovery periods are implemented, the order of the 2 training modalities becomes less critical. This is because it promotes molecular adaptive mechanisms after each type of training and reduces antagonistic responses to the 2 adaptations.^[66,67]^

## 6. Interval time between resistance and endurance training

The interference phenomenon observed in concurrent training can be influenced by the timing of endurance training relative to resistance training.^[5]^ Two hypotheses have been proposed to explain this interference phenomenon.^[4]^ One hypothesis suggests that endurance training within the same training session interferes with improvements in muscular fitness-related measures, while the other hypothesis posits that there is no interference effect when both training modes are performed in the same session. Several factors, such as exercise type, training load, exercise order, and the characteristics of the intervention population, contribute to determining which hypothesis holds true. Among these factors, the interval time or recovery period between the 2 training modes plays a significant role. When 1 training stimulus immediately follows another or occurs after a short interval, localized or generalized fatigue resulting from acute exercise can negatively affect the quality of the subsequent training.^[68,69]^

Earlier research by Sale et al demonstrated that a concurrent training program with a 24-hour recovery period between resistance and endurance training resulted in greater gains in maximal strength compared to performing both forms of exercise on the same day.^[70]^ Subsequent studies have supported the idea that properly separating endurance training from resistance training and increasing the interval between the 2 forms of training to allow for adequate recovery from exercise-induced fatigue leads to greater adaptations in endurance levels, muscle hypertrophy, and muscle strength.^[44,71]^

In the study of concurrent training on athlete, it has been suggested that to maximize muscle fitness adaptations, an ideal recovery interval of 3 to 6 hours between the 2 types of exercise should be implemented.^[72]^ Conversely, if the goal is to maximize endurance adaptations, a recovery interval of 24 hours between different exercise intensities should be employed.^[36,54,73,74]^ This approach helps prevent overlapping activation of myofibrillar and mitochondrial protein synthesis molecules, resulting in greater physiological adaptations in both training modalities.

Robineau et al conducted a study to investigate the effects of concurrent training with varying recovery times between the 2 training modes.^[54]^ After a 7-week intervention, it was observed that the development of strength qualities was hindered in the concurrent training group compared to the resistance training group alone. Moreover, the interference effect was more pronounced with shorter recovery times between the 2 training modes. This suggests that the relationship between resistance and endurance training is not straightforward, particularly in the context of high-intensity endurance training. To achieve optimal neuromuscular and endurance adaptations in high-intensity endurance training, it is recommended to have a recovery interval of at least 6 hours. In certain cases, it may be necessary to separate the 2 training modalities and allow for 2 full days of recovery to attain the highest level of adaptations.^[75]^

The appropriate time interval between 2 training sessions depends on the fatigue induced by the first training modality. Higher training loads lead to greater levels of fatigue, which requires longer recovery intervals between the 2 training modes. It has been observed that if resistance training is done before endurance training, the interval between the 2 sessions may need to be more than 48 hours, especially for women who may experience higher levels of fatigue compared to men.^[49,76,77]^ Therefore, when time allows, it is beneficial to schedule a specific interval between resistance and endurance training in order to optimize adaptations in both endurance and muscular fitness. However, more research is needed to further investigate the optimal time intervals for concurrent training and provide clearer guidelines.

## 7. Nutritional intervention

The effectiveness of a training program is often evaluated by observing athletic performances and physiological adaptations. While factors such as the training population, load, frequency, and timing are commonly considered in training program design, the importance of nutrition is sometimes overlooked. Nutrition plays a crucial role in the training process, and studies have shown that nutrient availability can significantly enhance training adaptations.^[78]^ For example, consuming low glycemic supplements during exercise can improve metabolism, enhance mitochondrial signaling responses,^[79,80]^ and promote the body’s adaptation to endurance training.^[81]^ Additionally, protein supplementation before and after exercise can enhance muscle protein synthesis.^[82,83]^ However, most studies on sports nutrition have focused on single exercise modes, and there is limited research on nutritional interventions during concurrent training.

To minimize the interference of concurrent training with strength adaptations, interventions that incorporate nutrition during training can help mitigate the interference effect. Protein plays a crucial role in promoting muscle growth and remodeling. Researchers have observed that muscle protein synthesis rates after protein supplementation during a single session of concurrent training were comparable to those seen during resistance training alone.^[84]^ In a study by Shamim et al, a high-protein diet and protein supplementation were implemented postexercise during a 12-week concurrent training program to investigate the long-term adaptation process and assess the impact of protein intake.^[85]^ The findings indicated that concurrent training combined with protein intake have similar improvements in maximal strength, lean body mass, and aerobic capacity compared to resistance training alone. This suggests that nutritional interventions involving protein intake during long-term concurrent training can mitigate the disruptive effects on muscle fitness.

For individuals aiming to maximize the benefits of concurrent training while minimizing interference, several strategies can be considered. These include imposing longer recovery periods of 6 to 24 hours between training modalities, limiting the frequency of endurance training to no more than 3 days per week, using a power bike for endurance training, and implementing postexercise nutritional interventions. By adopting these strategies, individuals can experience physiological adaptations that enhance both endurance and muscle fitness during concurrent training.

## 8. Gender differences

Within the current sports science research literature, there is a relative scarcity of studies focusing on various indicators among females. Moreover, there is a notable lack of studies specifically targeting females in the research on concurrent training. Instead, many studies extrapolate findings from research on males to females, which may not be appropriate. This is because there are considerable physiological and psychological differences between the 2 sexes, leading to distinct physiological adaptations during exercise.^[86]^

Physiologically, there are marked differences between males and females in terms of physical development and the oxygen transportation system. For instance, females experience the growth spurt during puberty approximately 2 years earlier than males. Males typically have a heart that is 10% to 15% heavier and 18% larger in volume than that of females, resulting in higher per-beat output and arterial blood pressure. Females generally have smaller thoraxes and weaker respiratory muscles, which leads to weaker respiratory function compared to males. Additionally, males tend to have greater aerobic capacity due to higher percentages of circulating blood in relation to body weight and increased hemoglobin levels. Regarding body composition and the musculoskeletal system, females typically have a higher subcutaneous fat content, approximately twice that of males, which can limit their exercise capacity. Conversely, males tend to have greater muscle content and heavier bones, approximately 10% heavier than those of females. Women also experience a higher rate of bone loss throughout their lifespan compared to men, resulting in lower average bone density.

These structural, morphological, and functional differences necessitate additional considerations for women when participating in certain sports and exercise routines. Research has shown significant differences in skeletal muscle adaptation and hormone secretion between males and females following concurrent resistance training at the same relative intensity.^[87]^ Recovery of skeletal muscle-related fitness indicators has been found to vary between males and females for up to 4 days after resistance training.^[88]^

Gender differences are also evident in resistance training effects. While muscle hypertrophy and lower limb strength adaptations appear similar between males and females, the reverse is observed for upper limb strength adaptations. In fact, females tend to exhibit greater adaptability in upper body strength compared to males, particularly when moderate-intensity resistance training is applied to untrained individuals.^[89–91]^

Additionally, women’s exercise capacity varies with their menstrual cycle phases, leading to regular fluctuations. Studies have revealed that women’s overall physical and aerobic capacity correlates significantly with their menstrual cycle phase, with the luteal phase showing the strongest correlation and the premenstrual and menstrual phases demonstrating the weakest.^[92]^ Given the substantial differences in physiological adaptations between males and females, it is essential to tailor training programs to gender-specific needs, whether for high-level athletes or the general population, in order to maximize training effectiveness.

## 9. Conclusion

In the field of concurrent resistance and endurance training, there is a complex landscape of physiological adaptations and training outcomes. Some studies suggest that concurrent training may reduce muscle hypertrophy and maximal strength compared to resistance training alone. However, a greater number of studies indicate that concurrent training does not diminish muscle fitness adaptations and even may enhance muscle hypertrophy. The concept of an “interference effect” in concurrent training is multifaceted and depends on various factors, making the theory somewhat ambiguous. One critical factor influencing the interference effect is the competence level of the individual undergoing training. The level of expertise and specificity of skeletal muscle molecular responses post-training likely play a pivotal role in determining the extent of interference during concurrent training.

It is worth noting that while many studies delve into the molecular mechanisms underlying muscle strength and hypertrophy adaptations in concurrent training, there is a need to shift focus towards exploring the practical application of these findings. This involves considering outcome indicators with significant effects and proposing strategies for their practical utilization. Many studies have primarily focused on mechanism exploration without addressing the substantial application value of their findings. Furthermore, the assessment of concurrent training effects relies on various indicators, including hormones and other sensitive markers. These markers can significantly impact the interpretation of training outcomes. In addition to training-related variables such as frequency, duration, intensity, volume, sequence, recovery time, and nutritional intake, non-training-related variables, such as evaluation methodology, also play a crucial role in elucidating the interference effect in concurrent training.

Future research endeavors have the potential to offer a more exhaustive comprehension of the multifaceted factors that govern neural and muscular adaptations during concurrent training paradigms. Consequently, this enhanced understanding can aid researchers and practitioners in devising training programs that are not only more effective but also personalized, thereby optimizing the benefits derived from concurrent training while mitigating interference effects. Furthermore, such investigative efforts can significantly contribute to the advancement of more precise and dependable assessment techniques, which are crucial for evaluating the efficacy of concurrent training interventions.

## Author contributions

**Conceptualization:** Shumin Bo.

**Investigation:** Tao Wang, Shumin Bo.

**Methodology:** Shumin Bo.

**Resources:** Tao Wang, Shumin Bo.

**Validation:** Tao Wang.

**Visualization:** Shumin Bo.

**Writing – original draft:** Tao Wang, Shumin Bo.

**Writing – review & editing:** Tao Wang, Shumin Bo.
